# A Culturally Congruent Psychosocial Intervention for Latino Caregivers of Children with Cancer: Formative Evaluation and Preliminary Efficacy

**DOI:** 10.3390/children13030392

**Published:** 2026-03-12

**Authors:** Michelle A. Fortier, Lessley Torres, Belinda Campos, Haydee Cortes, Sonia Morales, Carol Lin, Lilibeth Torno, Zeev N. Kain

**Affiliations:** 1 UCI Center on Stress and Health, School of Medicine, University of California-Irvine, Orange, CA 92868, USA; lessleyt@hs.uci.edu (L.T.); bcampos@uci.edu (B.C.); cortesh@hs.uci.edu (H.C.); zkain@hs.uci.edu (Z.N.K.); 2 Sue & Bill Gross School of Nursing, University of California-Irvine, Irvine, CA 92697, USA; 3 Department of Anesthesiology & Perioperative Care, University of California-Irvine, Irvine, CA 92697, USA; 4 Department of Chicano/Latino Studies, University of California-Irvine, Irvine, CA 92697, USA; 5 Department of Oncology, Rady Children’s Health, San Diego, CA 92123, USA; sonia.morales@choc.org (S.M.); clin@choc.org (C.L.); ltorno@choc.org (L.T.); 6 Department of Pediatrics, School of Medicine, University of California-Irvine, Irvine, CA 92697, USA

**Keywords:** social determinants of health, healthcare disparities, pediatric oncology, community-based participatory research, pediatric palliative care

## Abstract

**Highlights:**

**What are the main findings?**

- A community co-developed intervention to address quality of life in Spanish-speaking families impacted by childhood cancer was feasible, evidenced by high consent and adherence rates, and well-liked and well-received by families.
- The community co-developed intervention was associated with increases in emotional well-being, improvements in health literacy, and high satisfaction ratings.

**What are the implication of the main findings?**

- Effectively engaging with community members can result in a culturally congruent intervention that is well-received and relevant for Spanish-speaking families impacted by childhood cancer.
- The Corazones intervention was well-received and associated with improvements in quality of life outcomes for Latino families and can serve as a model for engaging community to address cancer health disparities.

**Abstract:**

**Background**: Parents of children with cancer experience significant psychological distress that is associated with poorer health outcomes. A recent review of caregiver interventions illustrated none targeting Latino parents of children with cancer and a significant need for culturally congruent intervention approaches. **Aims**: Following our first paper in this issue describing the development of a community co-developed intervention to address psychosocial outcomes in Spanish-speaking Latino families impacted by childhood cancer, this second paper describes the formative evaluation and exploratory analysis of preliminary efficacy in a single-arm pre–post trial. **Methods**: A total of 32 Spanish-speaking Latino parents/caregivers of children with cancer received the 12-session intervention targeting health literacy, culturally congruent care, and caregiver well-being. Quantitative measures of health literacy and emotional well-being were collected at baseline, post-intervention, and 3 months post-intervention and mixed methods formative evaluation data were collected immediately post-intervention. **Results**: Mixed methods formative evaluation showed that the intervention was useful, helpful, and relevant. Exploratory preliminary efficacy data using a non-parametric Friedman test showed that health literacy doubled from pre- (33%) to post-intervention (67%) and was sustained at 3 months (X^2^(2) = 12.52, *p* = 0.002; Cohen’s *d* = 0.65). Repeated measures ANOVA showed that emotional distress decreased significantly from baseline to immediately post-intervention with sustained treatment effects at 3 months post-intervention (*F*(2,62) = 4.37, *p* = 0.046; Cohen’s *d* = 0.42). Satisfaction scores were well above treatment acceptability (*M* = 39.13, *SD* = 2.80). **Conclusions**: Implementation of a community co-developed intervention with the goal of achieving cultural congruency was feasible, likeable, and relevant for Spanish-speaking Latino parents and caregivers of children undergoing treatment for cancer. Moreover, the exploratory analysis showed the intervention was associated with improvements in health literacy and emotional well-being and high levels of treatment satisfaction.

## 1. Introduction

Racial and ethnic disparities in cancer-related morbidity and mortality are largely associated with social determinants of health, such as disparities in economic status and access to quality healthcare [1]. The Latino population is the largest minoritized ethnic group in the United States (U.S.) [2] and cancer health disparities are persistent and pervasive in this group. Latino individuals have less access to state-of-the-art cancer treatment and personalized care and experience poorer health outcomes even into survivorship [3,4]. A recent review in the U.S. highlighted how the Latino population is one of the most disproportionately affected in a wide range of contributors to disparities, including low socioeconomic status, lack of access to insurance and integrated care, and a significantly higher current and rising incidence of acute lymphoblastic leukemia, the most common childhood cancer [5]. Social inequities contribute to stress and negative health among Latino caregivers, further exacerbating cancer health disparities [6,7]. Moreover, healthcare providers tend to underestimate the needs of Latino parents of children with cancer [8], which can lead to lack of communication of important diagnosis- and prognosis-specific information and contribute to challenges in decision making and access to relevant supportive interventions. Therefore, addressing factors contributing to disparities in caregiver well-being in Latino populations is a high priority.

Parents of children with cancer suffer significant psychological distress [9]. Sources of caregiver distress are numerous and include factors such as navigating a complex medical system, performing medical care at home, managing their child’s and their own well-being, and continuing to maintain household obligations while managing hospitalizations and illnesses [10]. Increased caregiver distress is associated with poorer caregiver health outcomes, including negative impacts on emotional and physical health as well as increased healthcare utilization [11]. Moreover, evidence suggests that social determinants of caregiver stress in pediatric oncology, such as lower health literacy and socioeconomic status, can impact access to supportive care and negatively impact children’s psychosocial outcomes during cancer treatment and beyond [12]. Thus, caregiver quality of life and well-being are an important target for intervention because of direct effects on parents/caregivers and indirect effects on children’s psychosocial functioning.

To effectively address the social factors that drive disparities among Latino families affected by childhood cancer, it is necessary to engage community experts including parents and caregivers of children diagnosed with cancer. In the first manuscript in this series, using a community-based participatory research (CBPR) approach, we collaborated with our target population to develop Corazones, a culturally congruent and sustainable intervention. This newly developed intervention directly tackles key social determinants of health, including limited financial resources, restricted access to healthcare, transportation barriers, and systemic racism and discrimination that constrain interventions by language and cultural context (providing an intervention that is culturally congruent and delivered by bilingual and bicultural interventionists that includes relevant cultural values). This intervention also leverages technology to address barriers in access to care by delivering the intervention synchronously using web-based video conferencing. Recent data support the use of technology to deliver psychosocial interventions for patients and families facing cancer [13,14].

**Figure 1:**
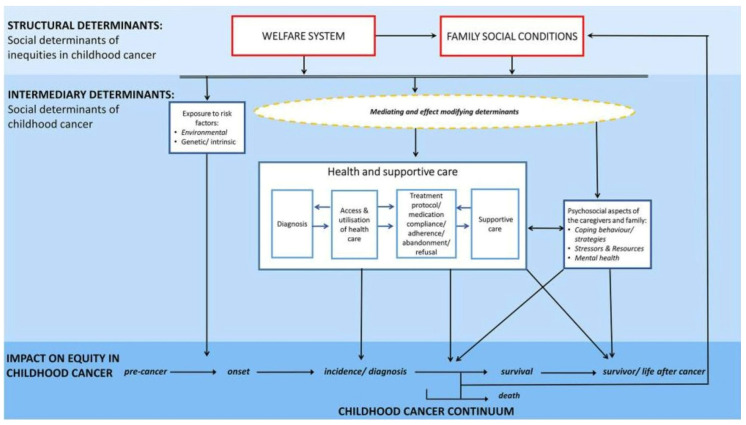
Conceptual Framework. Note. Framework created by Erdmann et al. [15].

The collaborative team (experts in psychology, behavioral science, oncology, implementation science, health equity, social work, nutrition, cultural adaptation, and community partners with lived experience) approached this work using the framework by Erdmann and colleagues [15], which highlights factors contributing to social inequities across the childhood cancer continuum, with a specific focus on supportive care and psychosocial factors within the family (Figure 1). The aim of this project was to address psychosocial factors related to supportive care in Figure 1 to strengthen and enhance coping strategies to promote quality of life.

The purpose of this second manuscript is to describe the formative evaluation process and outcomes, including evaluation of feasibility, likeability, and relevance of the intervention, as well as exploratory analysis of preliminary efficacy in a single-arm pre–post trial. The approach to intervention development was intentionally cancer diagnosis agnostic as we aimed to address significant gaps in access to psychosocial care by underserved families by targeting emotional well-being and health literacy independent of diagnosis. In addition, our aim was to ensure a culturally congruent approach to address this gap by integration of culturally grounded intervention strategies that incorporate cultural values and practices [16]. We hypothesized that the intervention would be feasible to implement, as evidenced by high rates of consent and engagement in intervention sessions, and that families who received the Corazones intervention would favorably rate the quality and relevance of the intervention components. We also hypothesized that the Corazones intervention would lead to improvements in health literacy and emotional well-being and that intervention satisfaction scores would be high.

## 2. Method

This study received Institutional Review Board approval, and all parents/caregivers provided informed consent. The Corazones intervention consists of 12 sessions that target three outcomes: health literacy, emotional well-being, and culturally congruent care. The intervention was delivered synchronously using web-based video conferencing in a group format. The sessions were delivered weekly for 12 weeks and all sessions were recorded and hosted on a web platform that allowed for asynchronous engagement if a participant could not attend synchronously.

The reader is referred to the first manuscript of this series for a detailed description of the Corazones intervention. Briefly, we engaged in community participatory-based research to partner with the community of focus—Spanish-speaking Latino families impacted by childhood cancer—to co-develop an intervention to improve quality of life during cancer treatment. This process resulted in a 12-session intervention targeting emotional well-being, health literacy, and culturally congruent care (intervention sessions detailed in Table 1). Given that cultural congruence stemmed from the incorporation of culturally relevant values into the intervention components, it was not assessed as a distinct variable.

### 2.1. Study Design

Consistent with the formative evaluation aim of this project, we implemented a single-arm pre–post trial. This approach allowed us to gather necessary feasibility and usability/likeability data which would support further evaluation of the Corazones intervention. In addition, we gathered outcome data related to our proposed intervention targets, including health literacy, emotional well-being, and treatment satisfaction. This was exploratory in nature as this study was not designed or powered to evaluate effectiveness.

### 2.2. Study Interventionists

Each interventionist was a community and/or hospital expert. We engaged bilingual and bicultural community experts in psychology, culinary medicine, CAM, and gardening to provide relevant sessions addressing emotional well-being, health literacy, and culturally congruent care. In addition, we engaged with bilingual and bicultural cancer center members to provide sessions on health literacy and spirituality. Based upon our intervention development we distilled each session content into component parts and developed fidelity checklists for each session that will be implemented in a future evaluation of the intervention.

### 2.3. Study Sample and Recruitment

**Figure 2:**
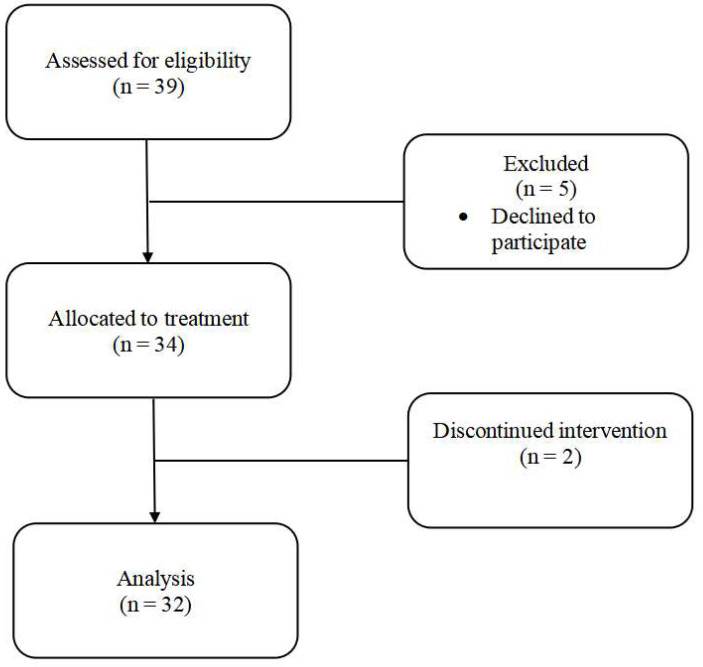
Participant Flow Diagram.

Parents and caregivers (e.g., grandparents, aunts, uncles, adult siblings, etc.) of children ages 2 to 17 years who were within 16 weeks of any cancer diagnosis were invited to participate in this study. We chose this age range given cancer in infants is rare and can have unique challenges to families. To limit each intervention group size to no more than 10 participants, we implemented the intervention in four consecutive groups. Eligibility criteria included that participants were Latino, reported Spanish as their primary language spoken (bilingual parents/caregivers were eligible to participate), and that their child was within 16 weeks of a cancer diagnosis. Parents of children with significant developmental disabilities or parents with severe psychiatric illness were excluded from recruitment. Given the need to recruit families engaged with the healthcare system, convenience sampling was used to approach all eligible families during the recruitment period. Recruitment was conducted between February 2022 and March 2023 but was not conducted when we were in the process of implementing the intervention. Over this time, a total of 39 caregivers were approached for this study and 34 (87%) consented to participate. Two caregivers discontinued participation due to complexities of their child’s treatment and thus 32 parents/caregivers (94%) participated in the majority of the sessions (80%) and completed all outcome assessments (See Figure 2).

### 2.4. Measures

#### 2.4.1. Formative Evaluation

Likeability and Relevance—Open-ended Interviews. All participants engaged in individual open-ended interviews to evaluate likeability and relevance of the intervention components. Participants were asked why they participated in the study, their thoughts on the components, which components were preferred and why, any changes they would like to see to the intervention, how the intervention impacted their functioning and family, etc.

Likeability and Relevance—Quantitative Evaluation. Post-intervention, quantitative measures of likeability and relevance were gathered using a 0–10 Likert-type scale. Participants were asked to rate each of the intervention components, with higher scores indicating more positive responses (very helpful or very useful).

#### 2.4.2. Preliminary Efficacy

Parent/Caregiver Health Literacy—The Newest Vital Sign. A primary target of the Corazones intervention is health literacy given documented disparities in health-related literacy among Latino caregivers, which is compounded by limited English proficiency (LEP), and cultural values that may be in conflict with U.S. healthcare systems/approaches [17]. Parent/caregiver health literacy was assessed using the Newest Vital Sign [18], a quick and efficient measure of health-related literacy that involves interpreting and applying data on nutritional labels and has been widely used as a measure of health literacy in diverse populations [19]. The NVS produces a total score from 0–6, with 0–1 reflecting limited health literacy, 2–3 possible limited literacy, and 4–6 reflecting adequate literacy.

Parent/Caregiver Emotional Distress—PROMIS short form 8a. There is a significant lack of culturally adapted interventions addressing parent distress in Spanish-speaking Latino caregivers. Accordingly, we expect our Corazones intervention to significantly reduce parent distress by addressing self- and family care via information provision and skills training. Parent/caregiver emotional well-being was measured using the PROMIS short form 8a [20] (nihpromis.org). The depression scale assesses 8 items of emotional stress and produces a standardized score.

Parent/Caregiver Treatment Satisfaction—Treatment Evaluation Inventory-Short Form. Parent/caregiver satisfaction is an important component of patient-centered care and a major determinant of hospital reimbursement [21]. Because the Corazones intervention is culturally congruent and incorporates convivial collectivist cultural elements [22], we expected that Corazones would improve satisfaction of parents and caregivers during cancer treatment because of the cultural congruence of the intervention. Parent/caregiver satisfaction was evaluated using the Treatment Evaluation Inventory-Short Form (TEI-SF) [23]. The TEI has been used to assess both parent and child satisfaction with interventions targeting a range of conditions in children, including cancer-related outcomes, and is a well-validated measure [23].

### 2.5. Procedures

Potential participants were identified in collaboration with oncology providers and by review of electronic health records (EHRs). Potentially eligible parents/caregivers were recruited in person during scheduled healthcare visits or via phone by a trained research associate fluent in Spanish to discuss the purpose of the study, eligibility, and study requirements. Participants were also assured that they were free to discontinue the study at any time, for any reason, and without any adverse effects. Following informed consent, participants completed study measures at baseline, post-intervention, and three months post-intervention using REDCap. Once baseline questionnaires were completed, participants were provided credentials to access our online intervention hosting platform. Only members of the research team and study participants have access to the site. Participants were consecutively enrolled until at least five caregivers were recruited to start implementation of the group intervention. Once the intervention started, sessions were scheduled on the dates and times most convenient for participants to encourage regular attendance. Meeting links were shared with participants on a weekly basis via text or email. All sessions were delivered in Spanish except for Spirituality (week 7), which involved the use of an interpreter. Because the goal of the Corazones intervention is to improve quality of life for the family and to be consistent with the cultural value of familismo, which prioritizes the needs of the family and family well-being, additional family members joined sessions at times; additional family members, however, did not complete study measures. Participation in the study required internet access; therefore, participants were provided computer tablets as well as mobile hotspots throughout the intervention period to ensure equal opportunity for participation in this virtual pilot intervention. A member of the research team was present at each intervention session to assist with any technical issue that could arise.

Immediately following the completion of the interventions, caregivers were scheduled to participate in an individual interview and were asked to complete the formative evaluation quantitative measures. Participants also completed the preliminary efficacy measures. Qualitative interviews were conducted via video conferencing by two Spanish-speaking research associates who were not involved in the delivery of the intervention. Each session was audio-recorded to allow for transcription and translation for analysis. Finally, the preliminary efficacy measures were sent to participants to complete via REDCap for the 3-month post-intervention assessment point.

### 2.6. Data Analysis

Qualitative Data. Analysis of qualitative data was guided by CBPR principles using modified grounded theory using inductive techniques and ongoing comparative analysis to ensure data saturation was complete [24]. Thematic analyses of likeability and relevance/helpfulness of each intervention component/session were conducted to identify potential modifications to the intervention. Textual information was coded using a computer-based system (Atlas/Ti). Data analyses were iterative because qualitative data collection and analysis occur simultaneously and concurrently [25]. Data were analyzed immediately using constant comparative analysis to systematically code the data [26]. A range of answers for each question were reviewed and discussed with the entire research team. Codebooks were generated, continually updated, and reviewed. Coding was conducted in multiple stages. First, a researcher conducted an initial familiarization of the data through repeated readings. Subsequently, text segments were assigned to predefined codes from the deductive codebook. Data that did not align with existing codes but appeared analytically significant were flagged and discussed to determine whether modification or expansion of the coding framework was warranted. To enhance reliability, a subset of the data was independently coded by a second researcher. Intercoder agreement was assessed, and discrepancies were resolved through discussion and consensus, leading to further refinement of code definitions where necessary. Frequency of coded references was recorded (i.e., a count of the number of times an identified theme was discussed by participants). We employed “member checking” by re-contacting randomly selected members of the interviews (60%, which achieved data saturation) for the purpose of confirming that the data analysis/interpretation made sense and reflected their perspective [27]. This process involved sharing transcripts and preliminary results of data coding and gathering feedback in order to clarify and confirm findings. Member checking confirmed the coding process.

Quantitative Data. Data analysis was performed using SPSS 27.0 (SPSS Institute, Chicago, IL, USA). Descriptive data regarding likeability/relevance is presented via median and interquartile range (IQR). To evaluate associations between the intervention and target outcomes, a non-parametric Friedman test was used for health literacy, given the positive skewness of the data, and repeated measures analysis of variance (ANOVA) was used for emotional distress to determine change from baseline to immediately post-intervention and sustainment at 3 months post-intervention. Finally descriptive statistics were used to present treatment satisfaction outcomes. Given the exploratory nature of the preliminary efficacy analysis on health literacy and emotional well-being, corrections for multiple comparisons were not made and are noted in the limitations section below.

## 3. Results

### 3.1. Participants

The 32 participants who completed the intervention were on average 40.09 ± 8.29 years of age and had 10.65 ± 3.66 years of education. Most participants were married (48.5%) or lived with their partner (21.2%). Average annual income was reported to be 32,390.00 ± 7312.81 U.S. dollars; however, only 12 participants reported income data and thus these numbers, which are only 28% of the median income for the geographical area and well below poverty level, may not represent the population [28]. Nonetheless, these data are consistent with income data reported in our first paper. Most (84.8%) participants were women, and the majority (68.8%) were from Mexico. Participant demographics are presented in Table 2.

### 3.2. Formative Evaluation—Qualitative Data

Thematic data of engagement and intervention likeability and relevance are presented in Table 3.

Session Engagement. Qualitative data revealed that participants valued session attendance, but the unpredictability of their children’s health needs and healthcare visit requirements impacted ability to attend. Nonetheless, participants often joined sessions in clinic settings, while traveling to appointments, during other activities occurring at home, and while sitting in their car in between commitments. All participants reported the virtual nature of session delivery facilitated engagement even if attendance was impacted by life circumstances. All participants also reported that having the intervention content hosted on the study website was a valuable way to revisit and engage with intervention materials, particularly when attendance was disrupted.

Likeability and Relevance. All sessions received positive feedback, although it was noted that the fitness component was the least useful, likely because it was difficult to engage in outside of dedicated intervention session time. In terms of potential intervention modifications, participants were asked about any potential burden from engaging in the intervention sessions and not only reported experiencing no burden but requested adding additional sessions including one additional CAM session (for a total of three) and one psychoeducation session (for a total of four), for a total of 14 sessions in the intervention moving forward. Caregivers also suggested a hybrid format, with the three CAM sessions, one of the two gardening sessions, and the live cooking session in person. Feedback also supported incorporation of an intake form prior to the Q&A sessions to gather family informational needs a priori to tailor the sessions.

Most families (88%) reported use of learned skills at home, particularly with CAM, culinary medicine, and gardening components and many (84%) reported that the intervention helped them incorporate self-care into their daily/weekly routine. Anecdotally, we observed unanticipated impacts of the intervention. These included reports from the clinical oncology providers of instances of organic patient navigation, in which caregivers who completed the intervention were observed to assist newly diagnosed Spanish-speaking families with social support, resources, and information. Finally, although not formally assessed, nearly all families (97%) reported in qualitative interviews that the intervention improved perceived social support and led to a decrease in feelings of isolation.

### 3.3. Formative Evaluation—Quantitative Data

Session Engagement. Engagement in sessions was high when examining session attendance both synchronously and asynchronously. Specifically, caregivers were 71% adherent with attending all 12 sessions synchronously and 97% adherent with engaging in all 12 sessions via the web platform that hosted all intervention content.

Likeability and Relevance. Two participants did not complete all quantitative measures of formative evaluation, resulting in differing denominators as noted below. When asked to rank sessions in terms of likeability, the psychoeducation sessions were rated as the top session by 40% of participants, CAM by 20%, culinary medicine by 20%, Q&A forums by 10%, and gardening sessions by 10%. Ranking by relevance/helpfulness revealed that 40% of participants rated psychoeducation as most relevant/helpful, CAM was ranked highest by 30% of participants, and Q&A by 30%. Descriptive results showed that participants were highly satisfied with the overall program (10.00 [10.00, 10.00], IQR = 0) and reported that it was highly relevant (10.00 [9.75, 10.00], IQR = 0.25), that the techniques learned were easy to implement at home (10.00 [9.00, 10.00], IQR = 1.00), and that, as a result of the intervention, they felt better prepared to navigate their child’s treatment (9.50 [0.0, 10.00], IQR = 10.00).

### 3.4. Preliminary Efficacy

Shapiro–Wilk tests of normality showed non-normally distributed scores for the NVS at baseline (*p* < 0.001) and post-intervention (*p* = 0.039) and trending at normal for 3 months post-intervention (*p* = 0.056). Conversely, Shapiro–Wilk tests of normality for the PROMIS-SF Depression Scale demonstrated normality of the data at baseline (*p* = 0.060), post-intervention (*p* = 0.566), and 3 months post-intervention (*p* = 0.136). Accordingly, the non-parametric Friedman test was used to analyze within subjects changes over time for the NVS data and ANOVA was utilized to examine within subjects effects across the intervention time points for the PROMIS-SF Depression Scale.

Preliminary efficacy data are presented in Table 4. Percent correct of items on health literacy as measured by the NVS doubled from pre- (33%) to post-intervention (67%) and was sustained at 3 months. Analysis of change in scores over time using repeated measures ANOVA demonstrated a statistically significant increase in health literacy scores from baseline to post-intervention and 3 months post-intervention (Cohen’s *d* = 0.65). In addition, depression scores on the PROMIS-SF Depression Scale decreased significantly from baseline to immediately post-intervention with sustained treatment effects at 3 months post-intervention (Cohen’s *d* = 0.42). Finally, average scores on the TEI-SF were well above treatment acceptability scores (min cut-off = 27) with a mean of 39.13 (*SD* = 2.80).

## 4. Discussion

Under the conditions of this pilot study, we demonstrated that implementation of an intervention that was co-developed with community members with the goal of achieving cultural congruency was feasible, likeable, and relevant for Spanish-speaking Latino parents and caregivers of children undergoing treatment for cancer. Moreover, this trial demonstrated feasibility of assessment of proposed intervention outcomes of health literacy and emotional well-being and was associated with improvements in each of the outcomes from baseline to post-intervention which were sustained at three months post-intervention. Although baseline depression scores were slightly elevated at baseline, they were not considered in the clinical range. Nonetheless, the changes in depression scores over the study were considered clinically significant. Moreover, the changes observed in health literacy over time indicate that families began with “possibility of limited health literacy” and moved to “adequate health literacy,” which was sustained over time. Although we observed statistically significant improvements in each outcome, it is important to note that, in the absence of a randomized controlled trial, we cannot definitively link these improvements to the Corazones intervention. Results of this study showed that families were highly satisfied with the intervention. We also achieved a high consent rate and high engagement in intervention sessions, despite the unpredictable and often hectic nature of families’ lives in the context of a child’s cancer diagnosis and subsequent demand of treatment regimens.

Participatory research approaches are growing more common and highlight the importance and effectiveness of engaging community partners in adaptation and tailoring of interventions for diverse populations. A recent review of interventions addressing racial and ethnic health disparities in the context of cancer included 10 studies that focused on Latino populations [29]. Approximately one-third of the studies utilized community-based participatory research in the intervention development/adaptation process; however, none of these interventions was focused on Latino parents/caregivers of children with cancer. Moreover, methods such as CBPR in the context of cultural adaptation, which involves developing an equal and collaborative partnership with the target community to develop interventions that meet community needs, provide an opportunity to integrate cultural context with evidence-based strategies to mitigate cancer health disparities. To our knowledge, the Corazones is the first intervention developed in collaboration with community members and delivered by community experts that is focused on quality of life in Spanish-speaking Latino families impacted by childhood cancer. Moreover, Corazones is a family-focused intervention which is important given recent findings of the need to integrate input of families to ensure psychosocial needs are met in interventions for pediatric health, particularly with a focus on health equity [30].

In addition to the positive associations we observed in the primary outcomes of health literacy and emotional well-being, we observed several unintended outcomes of the Corazones intervention anecdotally. Consistent with convivial collectivist culture, it was observed in the oncology setting by clinical providers that several families who received the intervention connected with newly diagnosed Latino families to provide support in navigating their child’s diagnosis and treatment. This phenomenon is likely related to cultural values of confianza, familismo, and personalismo. That is, embedding such cultural values within a psychosocial intervention may foster warm, trust-based relationships that validate families’ cultural identities and create psychologically safe spaces for vulnerability and mutual support. As parents and caregivers experience culturally congruent connection and dignity within the intervention, they may develop collective efficacy and a sense of communal responsibility. This relational foundation can naturally promote peer mentorship, positioning participating families to provide culturally responsive emotional and informational support to newly diagnosed families. Many approaches to mitigate health disparities in minoritized populations include patient navigators or community health workers and, in this regard, the Corazones intervention appeared to lead to this unintended but clearly important resource. Collaboration in intervention development and receiving the intervention also appeared to create a social support network that motivated families to support one another in clinic and hospital settings, as reported by families in the qualitative data assessment. This support was also evident in the work of the community partners who came together regularly to develop the intervention, such as via cafecitos and celebrations for one another, including a baby shower.

We acknowledge that an intervention such as Corazones that involves multiple components and multiple interventionists might raise questions of scalability and sustainability. Our next steps for the Corazones intervention is to conduct a large randomized controlled trial to evaluate effectiveness and in doing so we will use the RE-AIM framework [31,32,33] to guide this work. More specifically, as we evaluate effectiveness, we will simultaneously conduct a systematic evaluation of multiple factors associated with dissemination/implementation, via both quantitative outcomes and qualitative methods. We will also evaluate other efforts for dissemination and sustainability, such as by integrating services into routine standard care to minimize costs, cross-training personnel, and creating shared resources across cancer centers.

### 4.1. Limitations

In the context of the merits of this study, we also acknowledge its limitations. Although our primary aim was to evaluate feasibility, we also included exploratory analyses on outcomes of health literacy and emotional well-being. The use of a single-arm pre–post trial limits our ability to make causal conclusions about the impact of the intervention on the outcomes assessed. It is possible that changes in these outcomes were a function of natural changes over time or regression to the mean. Given our convenience sampling and lack of control group it is also possible our results reflect expectancy effects and/or social desirability bias. Most of our participants did not report income data, which limits generalizability and suggests our reporting of mean income may not accurately reflect our population. Our recruitment from a single site, exclusion criterion of severe psychiatric illness, and limited data on income limit our generalizability of findings and may have excluded some families most in need of resources. To address these limitations, our team is planning a fully powered randomized controlled trial to evaluate the Corazones intervention compared to an educational control condition. Finally, we acknowledge that the current pilot trial did not include formal evaluation of fidelity of intervention session implementation. The research team met with each of the interventionists to review study content and delivery following sessions, but we recognize this is insufficient to determine fidelity. For future evaluations of the Corazones intervention, we have developed fidelity checklists for each session. We will record all sessions and the research team who is independent from intervention delivery will review and code for fidelity.

### 4.2. Practical Implications

Results of this study can serve as a model for both engaging in a community co-developed intervention as well as assessing its feasibility and likeability/relevance. Results of the first paper in this series in combination with this outcomes paper can serve as a fruitful approach for successfully engaging with community partners, assessing community needs, and collaboratively developing and implementing a behavioral intervention to address gaps in care that may effectively mitigate cancer health disparities in minoritized and underserved populations. Partnering with communities to co-develop interventions requires a collaborative process over time in which power hierarchies are eliminated, lived experience is recognized as expertise, and community members have an equal “seat at the table” and are compensated for their engagement in the process. It also involves collaboratively disseminating results of this work both academically and within the community. Accordingly, it is important to note that our community partners chose not to co-author this publication with us and instead to focus efforts on community dissemination.

To support scalability of this intervention approach, cancer center providers and community partners were leveraged as interventionists, The compatibility with cultural norms and alignment with community priorities of our intervention support potential for community adoption. Accordingly, for future dissemination and to ensure access to this type of intervention for families experiencing children’s cancer treatment, we can partner with community healthcare settings to deliver the intervention to a broader population both locally as well as nationally, particularly using shared resources across cancer centers and benefiting from the virtual nature of the intervention. Sustainability of Corazones is supported by the virtual nature of intervention delivery which addresses barriers such as location and transportation. Moreover, many of our interventionists have begun to create virtual versions of their practices, which will lend further to increased sustainability in future implementation efforts and is particularly relevant to low-resource settings. We will also evaluate other efforts for dissemination and sustainability, such as by integrating services into routine standard care to minimize costs, cross-training personnel, and creating shared resources across cancer centers.

## 5. Conclusions

This two-part paper series describes the results of intervention development conducted by engaging collaboratively with community partners to create and evaluate Corazones, an intervention targeting health literacy, emotional well-being, and culturally congruent care for Spanish-speaking families impacted by childhood cancer. The first paper describes the process and intervention outcomes of equitably collaborating with community members. This paper describes formative evaluation results that support feasibility and usability of the intervention as well as preliminary efficacy results, which support positive improvements in health literacy and emotional well-being as well as high satisfaction ratings associated with intervention implementation. Future work on the Corazones intervention will involve effectiveness testing using an RCT design as well as evaluation of dissemination and implementation factors to support dissemination, including scalability and sustainability more broadly. This work demonstrates the benefits of the use of participatory research strategies to culturally adapt an intervention to be culturally congruent and address cancer health disparities for Latino caregivers and families affected by childhood cancer.

## Figures and Tables

**Table 1:** Intervention Description.

Session Description and Number of Sessions	Content	Target
Q&A—Oncologists (1)	Discussion of cancer-related care, decision making, clinically relevant information	Health literacy; culturally congruent care
Q&A—Social Work/Case Coordination (1)	Understanding disciplines and associated roles involved in care, psychosocial resources available	Health literacy, culturally congruent care
Culinary Medicine (2)	Understanding nutrition labels, culturally relevant ingredients, cancer-specific nutrition	Health literacy, culturally congruent care
Psychoeducation (3)	Emotion- and problem-focused coping, self-care, trauma-informed care	Emotional well-being, culturally congruent care
Complementary and Alternative Medicine (2)	Symptom management strategies for children including cancer-specific massage, acupressure, aromatherapy	Emotional well-being, culturally congruent care
Gardening (2)	Planting and caring for a salad bowl, maintaining and harvesting vegetables	Emotional well-being, culturally congruent care
Spirituality (1)	Identification of spiritual needs and connection with resources	Emotional well-being, culturally congruent care

**Table 2:** Participant Demographics.

Demographics	Parents (*n* = 32)
Age (yrs *M* ± *SD*)	40.09 ± 8.29
Sex (*n* (%))	
Female	27 (84.8)
Male	5 (15.6)
Education (yrs *M* ± *SD*)	10.65 ± 3.66
Partner Status (*n* (&))	
Married	15 (48.5)
Living Together	7 (21.2)
Other	10 (31.2)
Country of Origin	
Mexico	22 (68.8)
United States	6 (18.8)
Ecuador	3 (9.4)
Nicaragua	1 (3.1)

**Table 3:** Frequency of Coded References Across Themes.

Themes	Coded References	N of Coded References
		Total = 59
Health Literacy	Need to understand terminology related to diagnosis and treatment	20
	Need for information in practical terms that supports healthcare navigation	18
	Need for information in everyday language	21
Emotional Well-being		Total = 39
	Access to strategies to balance self- and family care in the context of cancer treatment	16
	How to support children’s adjustment during cancer treatment	13
	Access to resources and services (e.g., economic, social services) to support well-being	10
Culturally Congruent Care		Total = 58
	Integration of relevant cultural values into care	29
	Opportunities to gather health-related information in culturally congruent approaches	12
	Strategies for self-care that are culturally congruent and in Spanish	17

**Table 4:** Preliminary Efficacy Outcomes.

Variable	Baseline	IQR	Post-Intervention	IQR	3 Months	IQR	X^2^	*p*
Health Literacy (Median)	1.00	0–5	4.00	2–5.25	3.50	3–4.75	X^2^(2) = 12.52	0.002
Variable	Baseline	95% CI	Post-Intervention	95% CI	3 Months	95% CI	*F*	*p*
Depression (*M* ± *SD*)	16.72 ± 9.62	13.06, 20.39	13.35 ± 5.34	11.31, 15.38	13.79 ± 7.33	11.01, 16.58	*F*(2,62) = 4.37	0.046

Note. *M* = Mean; *SD* = Standard Deviation; IQR = Interquartile Range; CI = Confidence Interval.

## Data Availability

The original contributions presented in this study are included in the article, further inquiries can be directed to the corresponding author.
